# Analysis of familial exudative vitreoretinopathy (FEVR) cases in the UK 100 000 genomes project increases diagnostic rate and implicates heterozygous CTNND1 mutations in FEVR

**DOI:** 10.1136/jmg-2025-111083

**Published:** 2025-12-18

**Authors:** Dong Sun, Robert H Henderson, Emma Clement, Michel Michaelides, Angelos Kalitzeos, Genevieve A Wright, Eibhlin Mcloone, Chris Inglehearn, James A Poulter, Carmel Toomes

**Affiliations:** 1 Leeds Institute of Medical Research, University of Leeds, Leeds, UK; 2 Ophthalmology, Great Ormond Street Hospital for Children, London, UK; 3 Moorfields Eye Hospital NHS Foundation Trust, London, UK; 4 UCL, Great Ormond Street Hospital for Children, London, UK; 5 UCL Institute of Ophthalmology, University College London, London, UK; 6 Department of Ophthalmology, Royal Victoria Hospital, Belfast, UK; 7 Leeds Institute of Molecular Medicine, University of Leeds, Leeds, UK

**Keywords:** Eye Diseases, Genetic Variation

## Abstract

**Background:**

Familial exudative vitreoretinopathy (FEVR) is an inherited eye disease characterised by the incomplete development of the retinal vasculature. Over 10 genes have been associated with FEVR, but there are still a substantial number of genetically unsolved cases. The aim of this study was to analyse whole genome sequencing (WGS) data from the FEVR cases in the Genomics England (GEL) 100 000 genomes project to identify the causative variants.

**Methods:**

WGS was performed by GEL and accessed within the GEL Research Environment. FEVR cases were identified using LabKey and candidate variants were extracted using the ‘gene-variant workflow’ and ‘CNV/SV workflow’ and by using BCFtools in unfiltered VCF files.

**Results:**

Fifty-nine FEVR probands were submitted to GEL. We found six novel and eight previously reported pathogenic variants in six genes known to underlie FEVR (*TSPAN12, LRP5, FZD4, CTNNB1, KIF11* and *NDP*), as well as structural variants in *TSPAN12* and *KIF11*. These accounted for 15/59 (25.4%) of FEVR cases. We also found candidate heterozygous variants in *CTNND1* in three unsolved FEVR cases. Expanding the list of genes examined to include all genes reported to be mutated in ocular disorders likely solved a further four cases, indicating that these individuals may be misclassified as FEVR in GEL.

**Conclusion:**

By performing bespoke reanalysis of the FEVR GEL cohort, this study has highlighted additional heterozygous variants in *CTNND1* in FEVR cases and increased the diagnostic yield from 20% solved by the GEL analysis pipeline to 37% (22/59), but the majority of FEVR cases remain without a molecular diagnosis.

**WHAT IS ALREADY KNOWN ON THIS TOPIC**

- Studies in FEVR have identified mutations in eight genes as a cause of disease and a similar number of candidate genes, but 40%–60% of cases remain without a molecular diagnosis.

**WHAT THIS STUDY ADDS**

- This study analysed WGS data to try to increase the molecular diagnostic rate. The solved cases were almost doubled to 37% compared with the standard GEL pipeline, and variants in *CTNND1* were highlighted as a strong candidate gene of disease, but the majority of cases remained without a molecular result.

**HOW THIS STUDY MIGHT AFFECT RESEARCH, PRACTICE OR POLICY**

- This study suggests that the majority of FEVR cases are difficult to solve, even by WGS, and indicates that some pathogenic variants may be intractable to standard analyses.

## Introduction

 Familial exudative vitreoretinopathy (FEVR) is an inherited eye disease characterised by the abnormal and incomplete development of the retinal vasculature (Online Mendelian Inheritance in Man (MIM): 133780). The phenotype and penetrance of FEVR is highly variable; many mutation carriers have no noticeable visual problems and only display peripheral retina avascularity on examination, whereas a proportion of cases develop retinal ischaemia leading to secondary complications including neovascularisation, retinal folds and retinal detachment.^1^ FEVR is genetically heterogenous and the majority of molecularly solved cases contain pathogenic variants in one of eight genes: *NDP*, *FZD4*, *LRP5*, *TSPAN12*, *ATOH7*, *ZNF408*, *KIF11* and *CTNNB1*.^2^,^9^ However, variants in several other genes have been reported to underlie FEVR, but only single studies have been published to date, so their pathogenicity remains unconfirmed (*RCBTB1, ILK, JAG1, DLG1, TGFBR2, CTNNA1, CTNND1, EMC1, LRP6* and *SNX31*).^10^,^19^

The majority of FEVR cases with a molecular diagnosis have variants in genes which encode components of the Norrin-β-catenin pathway, in particular *FZD4, NDP, TSPAN12* and *LRP5*. This is a variant of the canonical Wnt-β-catenin pathway in which norrin (encoded by *NDP*) acts as a ligand (instead of Wnt) for a receptor complex composed of frizzled-4 (FZD4), low-density lipoprotein receptor-related protein-5 (LRP5) and chaperone protein tetraspanin-12 (TSPAN12). When norrin binds to the receptor complex, it halts the destruction of β-catenin (CTNNB1), causing it to accumulate in the cytoplasm and translocate into the nucleus to activate transcription of target genes.^20^ Interestingly, many of the unconfirmed candidate FEVR genes also have a connection with Wnt signalling and β-catenin (*JAG1*, *DLG1*, *CTNNA1*, *CTNND1*, *LRP6*, *EMC1*) but precise roles in the Norrin-β-catenin pathway remain to be deduced.^1521^,^25^

Despite the identification of pathogenic variants in many genes underlying FEVR, reports indicate that approximately 40%–60% of cases remain without a molecular diagnosis.^26 27^ The improved coverage offered by whole genome sequencing (WGS) has the potential to increase the solve rate in FEVR. Our study therefore focused on the analysis of the FEVR cohort in the 100 000 Genome Project (100KGP). The 100KGP contains data from around 100 000 individuals from families diagnosed with either a rare disease or cancer, recruited through UK hospitals within the NHS.^28^ All genomes have been sequenced, aligned and processed through the standard Genomics England (GEL) analysis pipeline, which identifies variants that pass standard minimum filters. These are then tiered based on family structure, frequency, gene-phenotype association and predicted impact, as previously detailed.^29^ Through the GEL research environment portal, researchers can access the WGS data of cases and recruited family members along with their clinical data recorded using Human Phenotype Ontology (HPO) terms. In addition, researchers can access the workflows, scripts and bioinformatics pipelines GEL provides to facilitate genomic research discoveries.

Here, we describe the re-analysis of WGS data from the FEVR cohort in the 100KGP in order to increase the molecular diagnostic rate, expand the mutation spectrum and identify new candidate FEVR genes.

## Methodology

### Participant selection

The FEVR cohort was extracted using R scripts from GEL LabKey Main programme portal (V.12) by setting the specific disease column as ‘Familial exudative vitreoretinopathy’. The clinical description (HPO) terms of these patients were extracted from the LabKey ‘rare disease phenotype’ dataset. 

### Variant analysis in known FEVR genes

Unfiltered VCF files were provided in GEL following alignment to human genome GRCh38 using Issac Genome Alignment Software and single nucleotide variant and indel calling using Starling software (https://github.com/sequencing/isaac_variant_caller). Variants in the reported FEVR genes were extracted using the ‘gene_variant_workflow’ script provided by GEL with configuration for Combined Annotation Dependent Depletion (CADD) scores added (https://cadd.gs.washington.edu/). Variants were further annotated using Variant Effect Predictor (VEP) (https://www.ensembl.org/info/docs/tools/vep/index.html) with plugins for spliceAI and UTRannotator.^30 31^ The exception to this was *NDP*, where variants were extracted from the GEL aggV2 database functional annotation VEP_105 folder. All variants were filtered to retain those with a minor allele frequency (MAF) <0.001 in the gnomAD (all population v3.1) (https://gnomad.broadinstitute.org/) and with either a CADD Phred score >15 or with a spliceAI score >0.2 or with predicted damaging effect through UTRannotator.

Copy number variations (CNVs) and structural variants (SVs) were called using Canvas (https://github.com/Illumina/canvas) and Manta (https://github.com/Illumina/manta), respectively, by using the GEL ‘CNV/SV workflow’ and SVRare.^32^ The variants were visualised and verified with Integrative Genomics Viewer (IGV) (https://igv.org/) and the breakpoints checked using Samtools (https://www.htslib.org/). The allele count of the selected pathogenic or likely pathogenic CNVs/SVs was accessed via SVRare in the GEL rare disease cohort (n=71 408).^32^

### Haplotyping

For pathogenic variants found in non-FEVR participants in GEL, the full participants’ list and clinical details were accessed through Participant Explorer and through R scripts in the LabKey portal. Haplotype mapping was performed using unfiltered VCF files annotated with VEP software using the v1.1 workflow provided in GEL. A 2-Mb region flanking the variants was selected using BCFtools (https://samtools.github.io/bcftools/bcftools.html) and variants with a gnomAD MAF between 0.01 and 0.1, with ‘PASS’ in the FILTER column and depth >=6, were selected for haplotyping using python scripts. The filtered VCF files were visualised in IGV and cross-checked in bam files to remove artefacts.

### Variants in other inherited retinal disorders (IRD) genes

The FEVR cohort was also checked for variants in 799 genes listed in the Gene2Phenotype eye gene panel (https://www.ebi.ac.uk/gene2phenotype) and in 692 genes listed in the GEL PanelApp Ophthalmological disorder panels (https://panelapp.genomicsengland.co.uk/) with all levels of evidence (final access: Feb 2023). SnpEff and SnpSift annotation and selection of variants with ‘PASS’ filter and with ‘Moderate’ to ‘High’ coding effects in unfiltered VCF files was applied with a similar strategy as described previously (https://pcingola.github.io/SnpEff/). Variants were then further annotated with ANNOVAR (https://annovar.openbioinformatics.org/) and filtered to retain variants with a MAF <0.001 in gnomAD (all population, v3.1) and predicted damaging effects with CADD (phred score >=20).

### Candidate gene searching in FEVR

Novel candidate gene identification in the unsolved FEVR cases was performed by first screening the de novo rare (MAF <0.001) coding region variants in the ‘de novo flagged variants’ dataset provided by GEL. For the unsolved FEVR trios not covered in this dataset, the de novo workflow was run with Platypus software (https://github.com/andyrimmer/Platypus/tree/master) with default settings and variants were further annotated with VEP. Finally, the SnpEff and SnpSift protocol was repeated for all genes on the unfiltered VCF files.

### Pathogenicity scoring

Variants were manually assessed using the Genoox Franklin website (accessed June 2023) for pathogenicity classification (https://franklin.genoox.com/clinical-db/home) according to the American College of Medical Genetics and Genomics (ACMG) guidelines.^33^ In addition, the frequency of all candidate variants identified was manually updated using gnomAD V4.1, and the SpliceAI scores were updated using SpliceAI lookup (https://spliceailookup.broadinstitute.org) (accessed Feb 2025). In this study, we considered variants to be disease causing if they had an ACMG score classification of ‘pathogenic’ or ‘likely pathogenic’. In addition, if the variant had an ACMG classification of variant of unknown significance (VUS) but additional information was available, either from cellular or animal model studies or from studies reporting further potentially pathogenic variants in the same gene, to suggest the variant could be disease causing, we assigned the proband ‘possibly-solved’.

## Results

### Variants in genes known to be mutated in FEVR

Sixty-four FEVR cases (59 probands and five affected relatives) were recruited to the 100KGP. Among them, 15 probands (25.4%) were found to have pathogenic variants in genes known to underlie FEVR: *TSPAN12*, *LRP5*, *FZD4*, *CTNNB1*, *KIF11* and *NDP* (table 1).

**Table 1:** Variants found in genes known to underlie FEVR in GEL FEVR cohort

ID	Participant type	Inheritance	Gene	Variant*	AC in GEL†	CADD‡	SpliceAI§	Genotype	gnomAD¶	ACMG**	Solved information	Reported information
1.1	Proband	Germline (affected mother)	*TSPAN12*	c.295del: p.(Ser99Valfs*8)	6	32	–	Het	6/1612560	LP	Solved by GEL	Novel
1.2	Proband’s affected mother	Unknown	*TSPAN12*	c.295del: p.(Ser99Valfs*8)	6	32	–	Het	6/1612560	LP	Solved by GEL	
3.1	Proband	Germline (unaffected father)	*TSPAN12*	Chr7 g.120754460-120807405del	9	NA	–	Het	Absent	LP	Solved by this study	Novel
10.1	Proband	Germline (unaffected father)	*FZD4*	c.1282_1285del: p.(Asp428Serfs*2)	5	32	–	Het	35/1613988	P	Solved by GEL	First described by^48^
12.1	Proband	Germline (unaffected mother)	*TSPAN12*	c.68T>G: p.(Leu23*)	2	38	–	Het	2/1612560	LP	Solved by GEL	First described by^6^
26.1	Proband	Biallelic (germline unaffected mother)	*LRP5*	c.2366C>A: p.(Ala789Asp)	2	27.1	–	Het	2/1613440	VUS	Solved by GEL	Novel
		Biallelic (germline unaffected father)	*LRP5*	c.2873G>A: p.(Arg958Gln)	7	29.2	–	Het	41/1614034	VUS	Solved by GEL	Novel
28.1	Proband	Biallelic (germline unaffected heterozygous parents)	*TSPAN12*	c.361–2A>G	6 (het:4, hom:2)	33	0.89 (acceptor loss)	Hom	Absent	P	Solved by GEL	First described by^49^
		*Germline (unaffected father*)	*LRP5*	*c.3890T>C: p.(Val1297Ala*)	*6(het:4, hom:2*)	*22.7*	–	*Het*	*1/1612746*	*VUS*	*This study*	*Novel*
32.1	Proband	Biallelic (germline unaffected heterozygous parents)	*LRP5*	c.4112–3C>G	4(het:2, hom:2)	23	0.5 (acceptor gain)	Hom	Absent	VUS	Likely solved by this study	First described by^50^
33.1	Proband	Germline (unaffected mother)	*NDP*	c.-70G>A	2	11.03	0.40 (donor gain)	Hemi	1/950031	VUS	Likely solved by this study	First described by^34^
37.1	Proband	Unknown (singleton)	*LRP5*	c.2237G>C: p.(Arg746Pro)	1	25.2	–	Het	Absent	VUS	Likely solved by this study	First described by^51^
41.1	Proband	Germline (affected mother)	*FZD4*	c.1478dup: p.(Met493Ilefs*42)	2	33	–	Het	Absent	LP	Solved by GEL	Novel
41.2	Proband’s affected mother	Unknown	*FZD4*	c.1478dup: p.(Met493Ilefs*42)	2	33	–	Het	Absent	LP	Solved by GEL	
44.1	Proband	Not from unaffected mother	*KIF11*	MantaDEL: chr10:92609838_92614346:DEL:−4508	1	NA	–	Het	Absent	LP	Solved by this study	Novel
		*Germline (unaffected mother*)	*LRP5*	*c.2116G>A: p.(Gly706Arg*)	*2*	*25.6*	*0.27* (*acceptor gain*)	*Het*	*2/1613922*	*LP*	*This study*	First described by^52^
50.1	Proband	De novo	*CTNNB1*	c.1420C>T: p.(Arg474*)	2	37	–	Het	Absent	P	Solved by GEL	First described by^53^
52.1	Proband	Germline (affected mother)	*FZD4*	c.1282_1285del: p.(Asp428Serfs*2)	5	32	–	Het	35/1613988	P	Solved by GEL	First described by^48^
52.2	Proband’s affected mother	Unknown	*FZD4*	c.1282_1285del: p.(Asp428Serfs*2)	5	32	–	Het	35/1613988	P	Solved by GEL	First described by^48^
53.1	Proband	Unknown (singleton)	*LRP5*	c.2317G>A: p.(Gly773Ser)	4	28.1	–	Het	31/1613350	VUS	Solved by GEL	Novel
57.1	Proband	De novo	*KIF11*	c.1727_1728del: p.(Ser576Cysfs*29)	1	26.1	–	Het	Absent	LP	Solved by GEL	Novel

Rows in italics are additional variants found in genes known to cause FEVR.

* Human Genome Variation Society (HGVS) annotation for transcripts *FZD4*, NM_012193.4; *KIF11*, NM_004523.4; *LRP5*, NM_002335.4; *NDP*, NM_000266.4; *TSPAN12*, NM_012338.4; *CTNNB1*, NM_001904.4.

† AC in GEL: total allele count seen in the whole GEL cohort.

‡ Scaled CADD scores of 20 means that the variant is among the top 1% of deleterious variants in the human genome, and a score of 30 means that the variant is in the top 0.1%.

§ SpliceAI score were provided with the max score between SpliceAI-acc-gain, SpliceAI-acc-loss, SpliceAI-don-gain and SpliceAI-don-loss.

¶ The minor allele frequency (MAF) of the variants was obtained from gnomAD version v4.1.0.

** American College of Medical Genetics and Genomics (ACMG).

CADD, Combined Annotation Dependent Depletion; FEVR, familial exudative vitreoretinopathy; GEL, Genomics England; LP, likely pathogenic; P, pathogenic; VUS, variant of unknown significance.

Four pathogenic or likely pathogenic variants were identified in *TSPAN12*. A novel heterozygous deletion spanning exons 7 and 8 of *TSPAN12* (NC_000007.14: g.120754460_120807405del) was detected in a sporadic FEVR proband (case 3.1) and their unaffected father. This deletion was absent from SV databases but was present in a further seven non-ophthalmic participants in GEL, who are presumably asymptomatic mutation carriers. All deletion carriers shared the same ethnicity and haplotype, suggesting that this deletion was inherited from a common founder (online supplemental figures S1, S2 and table S1). A novel heterozygous 1-bp deletion in *TSPAN12*, c.295del (p.(Ser99Valfs*8)), was identified in an FEVR proband and their affected mother (cases 1.1 and 1.2). Four further alleles of this variant were identified in the GEL cohort. Three of these cases were in the affected members of a family diagnosed with optic neuropathy and may represent a misdiagnosis. However, the fourth allele was found in an unaffected parent of a child with a non-ophthalmic diagnosis and presumably represents a case of non-penetrance. All the individuals were from the same ethnicity and shared a haplotype indicating it is a founder variant rather than a mutation hot spot (data not shown). Six heterozygous alleles of this variant were also reported in gnomAD in non-Finnish Europeans. Also in *TSPAN12*, a known heterozygous nonsense variant, c.68T>G (p.(Leu23*)), and a known homozygous splice acceptor variant, c.361–2A>G, were found in two further probands (12.1 and 28.1, respectively), and these alleles were both inherited from an unaffected parent.

Three FEVR probands were solved with *FZD4* variants. In two different cases (10.1 and 52.1), a previously reported heterozygous 4-bp deletion was identified, c.1282_1285del (p.(Asp428Serfs*2)). This variant was inherited from the affected mother of 52.1 and the unaffected father of 10.1. It was also present in an unaffected relative of a non-ophthalmic GEL participant. No shared haplotype was observed between these cases, indicating that it is a recurrent hotspot, consistent with the fact that this variant has previously been observed in multiple FEVR cases and 35 gnomAD individuals from different ethnicities. In FEVR case 41.1, and their affected mother, a novel heterozygous 1-bp duplication in *FZD4* was identified, c.1478dup, p.(Met493Ilefs*42).

Case 33.1 was considered likely solved with the discovery of a hemizygous VUS in *NDP*, c.-70G>A. This variant is in the 5’UTR and has previously been described as a cause of FEVR/Norrie disease in the NIHR-RD cohort.^34^ It has a SpliceAI score of 0.4 indicating it may cause mis-splicing of the transcript by introducing a splice donor site, but this remains to be experimentally verified.

*LRP5* variants were identified in four FEVR probands. Two cases harboured biallelic variants and two carried heterozygous changes. Case 26.1 had two novel heterozygous missense changes, c.2366C>A (p.(Ala789Asp)) inherited from their unaffected mother and c.2873G>A (p.(Arg958Gln)) from their unaffected father. The c.2366C>A variant was present in two individuals in gnomAD but was absent in GEL, whereas c.2873G>A was present in 41 individuals in gnomAD and in five additional GEL participants (two unaffected relatives of non-ocular disease, a cancer patient and a proband with mitochondrial disease and their father). Case 32.1 was found to have a homozygous splice acceptor variant in intron 19 of *LRP5* (c.4112–3C>G) which had previously been reported in a homozygous state in two affected members of a family with FEVR but was absent from all other datasets apart from the heterozygous unaffected parents. The effects of this variant have not been verified at the RNA level, but it generated a SpliceAI score of 0.5. A previously reported heterozygous *LRP5* substitution was identified in singleton case 37.1, c.2237G>C (p.(Arg746Pro)), which was absent from gnomAD and other GEL participants. A novel heterozygous change was identified in case 53.1, c.2317G>A (p.(Gly773Ser)). This variant was present in 31 alleles in gnomAD and in three additional GEL cases, all of whom had non-ophthalmic conditions or were unaffected relatives. Despite all these variants being classed as VUSs by ACMG, two of the cases were deemed solved by GEL, and the remaining cases harboured previously reported variants and are considered likely solved in this study. Two further *LRP5* missense variants were identified in cases 28.1 and 44.1, but these individuals harboured a second variant which was deemed to be the primary cause of disease (table 1).

**Figure 1:**
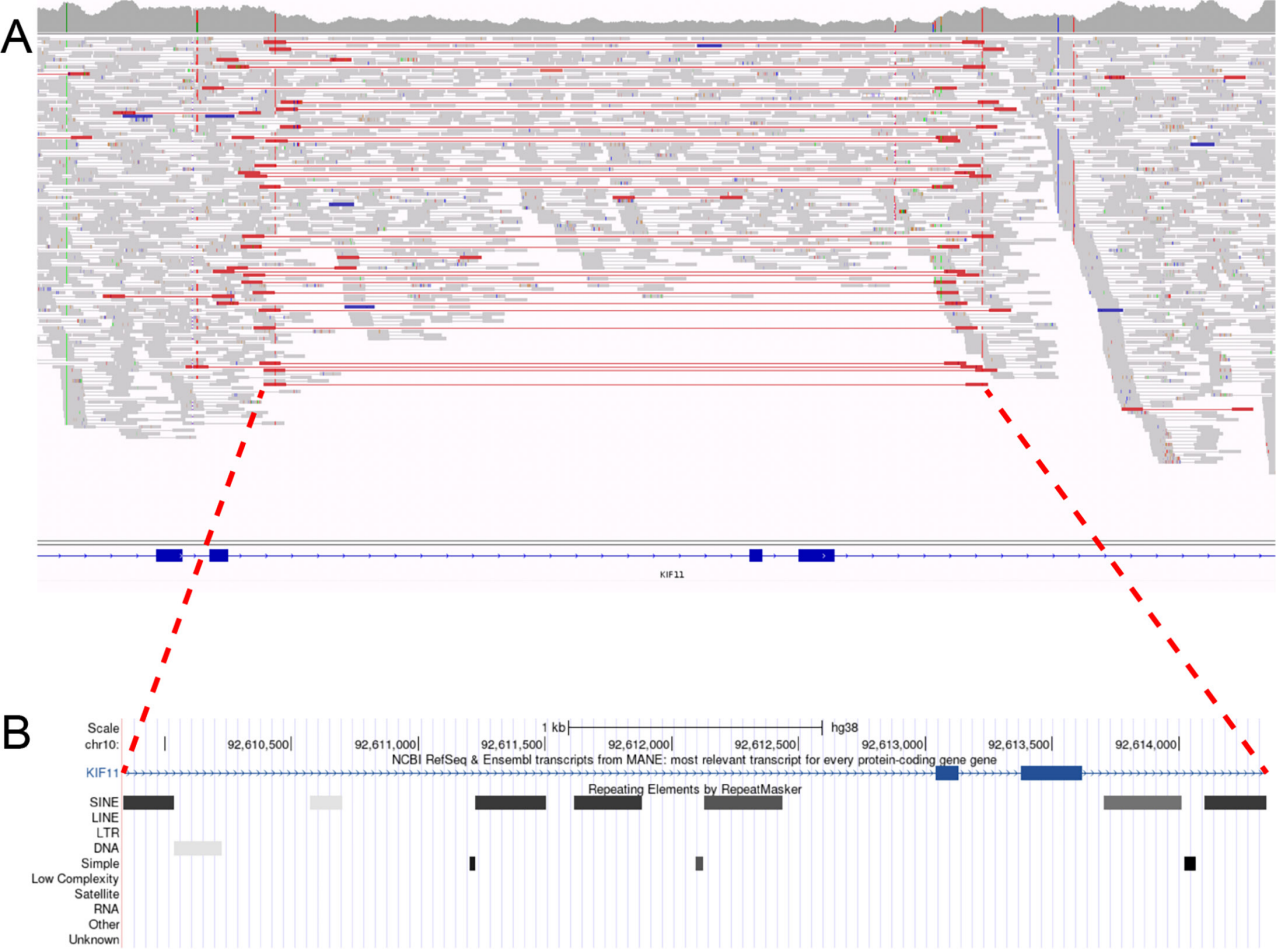
Deletion in KIF11 found in proband 44.1. (**A**) IGV visualisation of the SV. The precise breakpoints could not be determined due to highly similar repeat sequences flanking the deletion. (**B**) UCSC genome browser output showing chr10:92,609,838–92,614,346 (hg38), which is the deleted region defined by Manta. SINE repeats can be seen at either side of the region, which explains why precise mapping is not possible bioinformatically. IGV, Integrative Genomics Viewer; UCSC, University of California Santa Cruz; SINE, Short interspersed nuclear elements; LTR, Long terminal repeat elements; SV, Structural variant.

Two FEVR cases were solved with novel heterozygous variants in *KIF11*, a gene known to underlie FEVR with microcephaly. A large deletion spanning exons 7 and 8 of *KIF11* was identified in case 44.1 who had FEVR and microcephaly (figure 1). This deletion encompassed repeat sequences at the termini which prevented accurate annotation of the breakpoint, but according to Manta, it spans 4508 bp and is located at chr10:92609838–92614346 (hg38). This SV was not present in the unaffected mother, and the paternal DNA was not available for analysis. A 2-bp de novo deletion in *KIF11* was identified in case 57.1, c.1727_1728del (p.(Ser576Cysfs*29)), but microcephaly was not noted in the HPO terms. Both variants were absent from all other datasets.

A previously reported de novo nonsense variant was identified in *CTNNB1* in FEVR proband 50.1, c.1420C>T (p.(Arg474*)). This child only has ocular HPO terms recorded despite de novo variants in this gene typically causing a severe developmental syndrome including developmental delay, spastic diplegia and microcephaly. Indeed, a second GEL case with a phenotype matching the *CTNNB1*-deletion syndrome was reported as being solved with the same variant, although no eye phenotype was noted in this case.

### Variants in *CTNND1* in FEVR cases

**Figure 2:**
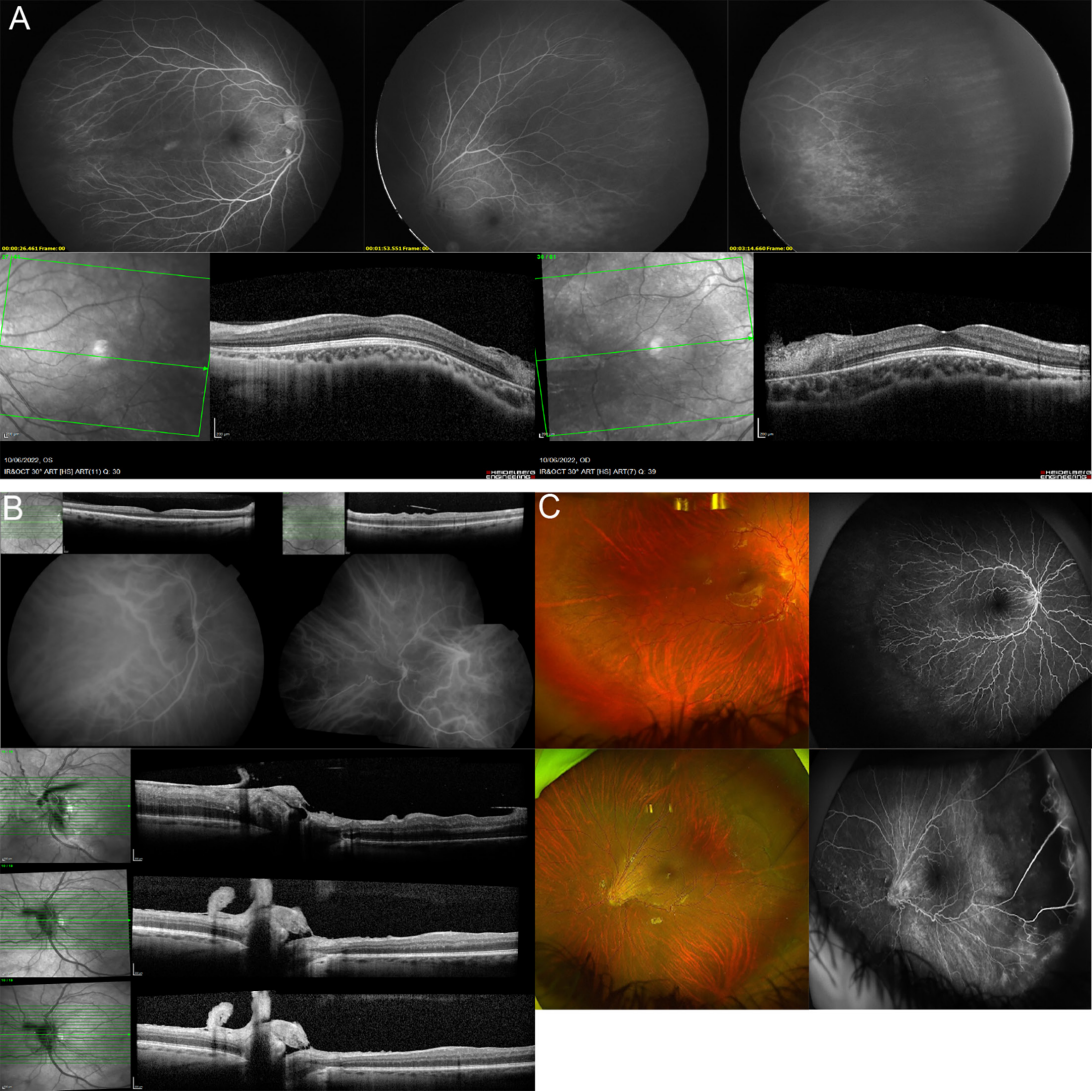
Clinical phenotype of FEVR caused by variants in CTNND1. (**A**) FFA and OCT photo of patient 49.1. The FFA images (upper panels) show the non-perfusion in the peripheral retina; the OCT images (lower panels) show the bitemporal hamartomatous lesions. (**B**) Asymmetrical vitreoretinopathy in patient 29.1, illustrated by asymmetrical vascular tortuosity, vessel straightening and vitreoretinal traction between eyes (upper panels). Longitudinal horizontal OCT scans (lower panels) through the left optic nerve head using the follow-up mode, showing a reduction in vascular tortuosity over time, with assessment at baseline, 5 years and 7 years. (**C**) Optos fundal images and oral fluorescein angiograms of patient 56.1 showing temporal and inferotemporal avascular retina with blood vessel tortuosity in the right eye (upper panel) and disc and macular dragging with peripheral temporal and inferotemporal pigmentary and vitreal changes, as well as temporal retinoschisis in the left eye (lower panel). FEVR, familial exudative vitreoretinopathy; FFA, fundus fluorescein angiography; OCT, optical coherence tomography.

Heterozygous variants in the candidate FEVR gene *CTNND1* were identified in three probands from the GEL cohort (table 2). A novel de novo splice donor variant was identified in case 49.1, c.1963+1G>T, which is predicted LP by ACMG. Fluorescein angiography (FA) revealed this child had the classic FEVR phenotype of bilateral peripheral retinal non-perfusion, along with likely hamartoma of both retinae and right temporal retinal scarring (figure 2A). In addition to this ocular phenotype, the proband had dermatofibrosarcoma protuberans and extensive dermal melanocytosis.

**Table 2:** Variants found in *CTNND1* in GEL FEVR cohort

ID	Participant type	Inheritance	Gene	Variant*	AC in GEL†	CADD‡	SpliceAI§	Genotype	gnomAD	ACMG**	Solved information	Reported information
29.1	Proband	Unknown (singleton)	*CTNND1*	c.935C>T: p.(Ser312Phe)	1	22.7	–	Het	4/1492894	VUS	Possibly solved by this study	Novel
49.1	Proband	De novo	*CTNND1*	c.1963+1G>T	1	35	1(donor loss)	Het	Absent	LP	Solved by this study	Novel
56.1	Proband	Germline (unaffected mother)	*CTNND1*	c.259G>A: p.(Gly87Arg)	2	28.6	–	Het	10/1613830	VUS	Possibly solved by this study	Novel

* Human Genome Variation Society (HGVS) annotation for transcript *CTNND1* is NM_001085458.2.

† AC in GEL means the total allele count seen in the whole GEL cohort.

‡ Scaled CADD scores of 20 means that the variant is among the top 1% of deleterious variants in the human genome, and a score of 30 means that the variant is in the top 0.1%.

§ SpliceAI score were provided with the max score between SpliceAI-acc-gain, SpliceAI-acc-loss, SpliceAI-don-gain and SpliceAI-don-loss.

¶ The minor allele frequency (MAF) of the variants was obtained from gnomAD v4.1.0.

** American College of Medical Genetics and Genomics (ACMG).

CADD, Combined Annotation Dependent Depletion; FEVR, familial exudative vitreoretinopathy; GEL, Genomics England; LP, likely pathogenic; P, pathogenic; VUS, variant of unknown significance.

A novel heterozygous *CTNND1* VUS was identified in singleton FEVR case 29.1, c.935C>T (p.(Ser312Phe)). This variant was found only in the proband in GEL and in 4/1492894 alleles in gnomAD and had a CADD score of 22.7. Fundus examination showed that this case had bilateral avascularity and retinal vessel tortuosity in their peripheral retina plus a longstanding vasoproliferative tumour in their left eye, previously treated with cryotherapy and panretinal photocoagulation (figure 2B). This individual also has type 1 diabetes mellitus and arthritis.

Another novel heterozygous *CTNND1* VUS was identified in case 56.1 and their unaffected mother, c.259G>A (p.(Gly87Arg)). This variant had a CADD score of 28.6, was not present in any other GEL participants and was in 10 individuals in gnomAD. Fundus examination showed bilateral temporal non-perfusion and tortuosity of the retinal blood vessels and left disc dragging with retinoschisis temporally (figure 2C). Additionally, this individual has mild to moderate learning difficulties. Due to the rarity of these missense variants, the conservation of the affected amino acids within homologues and their pathogenic prediction scores (online supplemental figure S3 and table S2), we considered these cases as strong candidates for being disease-causing, and therefore classified them as possibly solved in this study.

In addition to the variants described above, VUSs were detected in *CTNNB1* and *ZFN408* and in four genes reported in single studies to underlie FEVR (*ILK*, *EMC1*, *SNX31* and *DLG1*) (online supplemental table S3). Although these variants were identified in genes previously reported in association with FEVR, the available evidence for pathogenicity of FEVR causing variants in these genes remains tentative at this time, with only single studies for each reporting variants classified as VUS by ACMG. Thus, these cases were still considered as unsolved in this study.

### Variants in other IRD genes and novel genes

To determine if variants in other genes mutated in ocular disease were responsible for the phenotype in patients diagnosed with FEVR, the gene panel was expanded to include all known ophthalmic disorder genes. Four probands (6.8%) were found to have variants which likely accounted for their phenotypes (table 3). Cases 8.1 and 31.1 both contained biallelic variants in *USH2A*, while cases 17.1 and 20.1 had heterozygous variants in *PAX2* and *PRPH2,* respectively.

**Table 3:** Variants in other eye disease candidate genes found in FEVR patients

ID	Inheritance	Gene	Variant*	Genotype	gnomAD†	CADD‡	SpliceAI**	ACMG§	AC in GEL¶	Solved information	Reported information
8.1	Assumed biallelic (not from unaffected mother)	*USH2A*	c.1546G>A: p.(Gly516Arg)	Het	Absent	25.8	–	VUS	1	Solved by GEL	First described by^54^
	Assumed biallelic (germline from unaffected mother)	*USH2A*	c.1226G>A: p.(Trp409*)	Het	5/1 613 146	36	–	P	4	Solved by GEL	First described by^55^
17.1	Unknown (single case)	*PAX2*	c.365G>T: p.(Gly122Val)	Het	Absent	28.4	–	LP	1	Likely solved by this study	Novel
20.1	Unknown (single case)	*PRPH2*	c.828+3A>G	Het	Absent	20.9	0.58 (donor gain)	VUS	1	Solved by GEL	Novel
31.1	Assumed biallelic (not from unaffected mother)	*USH2A*	c.6049+2T>A	Het	4/1 613 826	31	1(donor loss)	LP	1	Likely solved by this study	Novel
	Assumed biallelic (germline unaffected mother)	*USH2A*	c.8132G>A: p.(Ser2711Asn)	Het	164/1 614 046	23.4	–	VUS	12	Likely solved by this study	Novel

* Human Genome Variation Society (HGVS) annotation for transcripts *USH2A*, NM_206933.4; *PAX2*, NM_000278.5; and *PRPH2*; NM_000322.5.

† The minor allele frequency (MAF) of the variants was obtained from gnomAD V4.1.0.

‡ Scaled CADD scores of 20 means that the variant is among the top 1% of deleterious variants in the human genome, and a score of 30 means that the variant is in the top 0.1%.

§ American College of Medical Genetics and Genomics (ACMG).

¶ AC in GEL means the allele count of the variant in the whole GEL cohort.

** SpliceAI score were provided with the max score between SpliceAI-acc-gain, SpliceAI-acc-loss, SpliceAI-don-gain and SpliceAI-don-loss.

CADD, Combined Annotation Dependent Depletion; Het, Heterozygous; LP, likely pathogenic; P, pathogenic; VUS, variant of unknown significance.

The full genomic dataset for all remaining unsolved FEVR cases was examined to try and identify new, previously unreported genes with pathogenic variants which may account for the FEVR phenotype. However, no variants/genes were common to more than one case with enough evidence to warrant further investigation. Therefore, following our extensive analysis, 37/59 FEVR cases (62.7%) remain without a molecular diagnosis.

## Discussion

In this study, we have analysed the FEVR cohort in the 100KGP with the aim of identifying new variants and genes involved in this disorder. Our researcher-led analysis has solved 15/59 (25.4%) cases and likely or possibly solved seven additional cases (11.9%), bringing the total number of solved/likely/possibly-solved cases to 22/59 (37.3%). The standard GEL pipeline had previously been applied to this dataset and solved 12/59 cases (20.3%) (tables1  3), so this bespoke analysis has almost doubled this figure, an uplift which is consistent with similar studies.^35 36^ This increase was due to the automated GEL pipeline missing: two SVs in key genes (*TSPAN12* and *KIF11*), three variants in genes underlying FEVR that were not tiered but have been reported as pathogenic in peer-reviewed studies, three variants in a gene recently and tentatively reported in FEVR (*CTNND1*) and two cases with variants in genes implicated in other non-FEVR eye diseases.

Four of the FEVR probands in the cohort were solved with variants in non-FEVR IRD genes, and it is unclear if these are cases of overlapping phenotype or if the disease classification in GEL is incorrect (table 3). Two individuals had biallelic variants in *USH2A* and a third carried a heterozygous variant in *PRPH2*. These genes are commonly associated with the ocular phenotype of retinitis pigmentosa (RP) (MIM: 268000). While the phenotypes for RP and FEVR would appear to be very different, retinal detachments can be observed in RP and 5% of RP cases are reported to have coats-like exudative vitreoretinopathy, which overlaps with the FEVR phenotype.^37 38^ Furthermore, a link between RP and FEVR exists as two of the genes reported to underlie FEVR, *ZNF408* and *RCBTB1*, have also been associated with RP.^39 40^

Case 17.1 was found to have a heterozygous novel missense variant in *PAX2*, c.365G>T, p.(Gly122Val) (table 3). Heterozygous variants in *PAX2* are known to cause papillorenal syndrome (MIM: 120330), a dominant disorder characterised by optic nerve dysplasia and renal hypodysplasia.^41^ This missense mutation is predicted as ‘likely pathogenic’ and is located in the paired domain of PAX2, where a number of other pathogenic missense mutations are located. No kidney defects were noted in case 17.1, but subclinical renal abnormalities have previously been reported in other cases of papillorenal syndrome.^41^ The retinal phenotypes reported in papillorenal syndrome can be widely variable and often involve retinal vasculature abnormalities and retinal detachment secondary to optic nerve dysplasia, so this syndrome could resemble FEVR.^41^ This variant is therefore likely to be causative for the phenotype observed in this case, but further clinical investigations need to be undertaken, especially kidney function tests.

Three FEVR probands were solved/possibly solved by variants in *CTNND1*. This gene encodes delta catenin (also known as p120) and heterozygous pathogenic variants in this gene are known to cause Blepharocheilodontic syndrome 2 (BCDS2) (MIM: 617681). BCDS2 is characterised by eyelid anomalies, facial dysmorphology (including cleft lip and palate) and tooth abnormalities, but a variety of additional features have also been described in the literature including cardiac and neurodevelopmental defects.^42^ However, a recent publication reported that variants in *CTNND1* can also cause FEVR.^16^ Three heterozygous *CTNND1* variants were reported in three Chinese families: c.949C>T (p.(Arg317Cys)), c.1867A>T (p.(Lys623*)) and c.2099G>A (p.(Arg700Gln)). All these cases had eye phenotypes consistent with a diagnosis of FEVR, but additional features of cleft lip/palate and syndactyly were noted in the members of the family with the nonsense variant. None of the individuals with *CTNND1* variants in the current study had reports of any extraocular features previously reported in *CTNND1*-related disease, and the additional phenotypes of type 1 diabetes and arthritis, found in proband 29.1, and dermatofibrosarcoma protuberans and dermal melanocytosis, reported in case 49.1, could be unrelated to the *CTNND1* variants. Re-examination of these three FEVR cases should be undertaken to rule out the presence of additional features, but if these diagnoses are correct, this suggests that the FEVR-associated variants may be hypomorphic and result in milder disease. The majority of the *CTNND1* variants which cause syndromic disease are de novo frameshift variants and are likely to result in nonsense-mediated mRNA decay (NMD), whereas the FEVR-related variants are predominantly missense substitutions (online supplemental figure S4).^42^ The exception to this observation is the de novo splice site variant identified in case 49.1 in intron 12. The most common outcome for splice donor mutations is skipping of the preceding exon, and while almost all *CTNND1* transcripts contain exon 12, it is an in-frame exon. This variant may therefore cause a deletion of 23 amino acids rather than NMD. However, additional experimental work is required to confirm the outcome of this variant. Nevertheless, these new FEVR cases add further evidence to support the hypothesis that variants in *CTNND1* cause FEVR.

As well as the variants in *CTNND1*, several mutations were found in other candidate FEVR genes (*ILK*, *EMC1*, *SNX31* and *DLG1*) in our study. However, the available evidence to date for pathogenicity of FEVR causing variants in these genes remains tentative. For example, three missense variants have been reported in *ILK* previously in FEVR,^11^ but all are classified by ACMG as VUS. Our study identified one additional VUS missense variant p.(Met301Val) in *ILK*. Even though functional knockout studies in animal models support a role for *ILK* in retinal angiogenesis,^11^ the human genetic evidence remains limited to date. Thus, the evidence accumulated from a previous study and from our current analysis remains insufficient to consider the case solved. By similar criteria, the VUSs identified in *EMC1*, *SNX31* and *DLG1* to date (online supplemental table S3) are notable but are again considered tentative for the purposes of this study. Thus, these cases were still considered as unsolved.

Despite this increased solved rate, the majority of the FEVR cases in the cohort remained without a molecular diagnosis, 37/59 (62.7%). This is not due to the presence of VUS in the known and candidate FEVR genes (online supplemental table S3), as including these would only solve a further seven cases, still leaving 30/59 (50.8%) unsolved. It is possible that VUS in non-FEVR IRD genes may account for some of the unsolved cases, and pathogenic monoallelic variants in genes known to underlie recessive IRD were identified in this cohort. For example, two unsolved FEVR cases had heterozygous pathogenic variants in *EYS* (which underlies recessive RP); case 22.1 carried a nonsense variant (NM_001142800.2 c.T8111G: p.(Leu2704*)), and case 46.1 had a duplication spanning exons 3–8 (NC_000006.12 g.65377012_65522370dup). However, no second allele could be found in either case and given that 36% of the population are reported to be carriers of recessive pathogenic IRD variants, these findings could be anecdotal.^43^

The level of phenotypic data supplied for the FEVR cohort in GEL is highly variable. The range of HPO terms submitted with each case varies between 1 and 15, with an average of 5.7 per case. Comparing the HPO terms between the solved and unsolved cases showed no major difference (online supplemental figure S5). Furthermore, inconsistencies in the use of HPO terms in the 100KGP have been reported elsewhere.^44^ Pressure to recruit from busy NHS clinics sometimes led to phenotypes being described using only one or two terms from one organ system, which could further reduce diagnostic success through misclassification in some cases. This therefore remains a limitation of this and any other 100KGP study and may act as a confounding factor in case classification.

In addition, it is possible that low penetrant variants with a higher MAF, similar to the *ABCA4* variant c.5603A>T, (p.(Asn1868Ile)) found in 7% of Europeans, might be excluded during the variant prioritisation.^45^ Alternatively, non-coding variants may have been missed as annotating these is still challenging, despite the introduction of tools such as UTRannotator and SpliceAI. The discovery of multiple non-coding SV at the RP17 locus provides an example of non-coding variants that may be overlooked using the current pipeline.^46^

There is also increasing speculation that some forms of FEVR may be caused by oligogenic inheritance and this hypothesis is supported by the large number of asymptomatic mutation carriers that are reported in FEVR and by observations in this study. Anecdotal reports of variants in more than one gene encoding components of the Norrin-β-catenin pathway have been reported, but these are often hard to confirm due to small sample sizes.^47^ In this study, rare potentially pathogenic variants were found in two cases in addition to the primary pathogenic variants (table 1). These variants were found during the mutation discovery pipeline, so they were identified using very stringent criteria. It would be interesting to widen this screen to less rare variants and perform comparison studies between cases, controls and asymptomatic mutation carriers. The identification of multiple individuals in this study with pathogenic FEVR variants but no reported ocular phenotype highlights how large datasets like 100KGP provide an ideal opportunity to identify increasing numbers of asymptomatic mutation carriers to aid in the identification of additional variants or genetic modifiers.

In conclusion, in this study, we increased the solve rate of FEVR cases in the 100KGP from 20.3% (12/59) to 37.3% (22/59) and highlighted heterozygous variants in *CTNND1* as further evidence that variants at this gene are a possible cause of FEVR. However, 62.7% of the cohort remain without a molecular diagnosis. This highlights the broader challenges in identifying pathogenic variants causing human diseases and the need for additional screening to identify non-coding variants, structural variants and complex rearrangements, as well as investigation of potential oligogenic and epigenetic mechanisms, which will require larger-scale genomic studies or international collaboration.

## Supplementary material

10.1136/jmg-2025-111083 online supplemental file 1

## Data Availability

All data relevant to the study are included in the article or uploaded as supplementary information.
