# Supplementary material for: Analysis of familial exudative vitreoretinopathy (FEVR) cases in the UK 100 000 genomes project increases diagnostic rate and implicates heterozygous CTNND1 mutations in FEVR
Source: J Med Genet. 2025 Dec 18;63(3):e111083. doi: 10.1136/jmg-2025-111083 (PMC13018757; doi:10.1136/jmg-2025-111083)
Supplement: online supplemental file 1 [file jmg-63-3-s001.pdf]

Analysis of familial exudative vitreoretinopathy (FEVR) cases in the UK 100,000 genomes project increases diagnostic rate and implicates heterozygous CTNND1 mutations in FEVR.

Supplementary Data

***Figure S1.* Common *TSPAN12* exon7-8 deletion identified in nine GEL participants.** (A) Split view visualization of the deletion in all nine GEL participants in IGV. (B) Samtools soft-clipped read mapping visualized in IGV identified the same breakpoint in all individuals with the *TSPAN12* exon7-8 deletion, chr7:120,754,460-120,807,405 (hg38) size=52,945 bp.

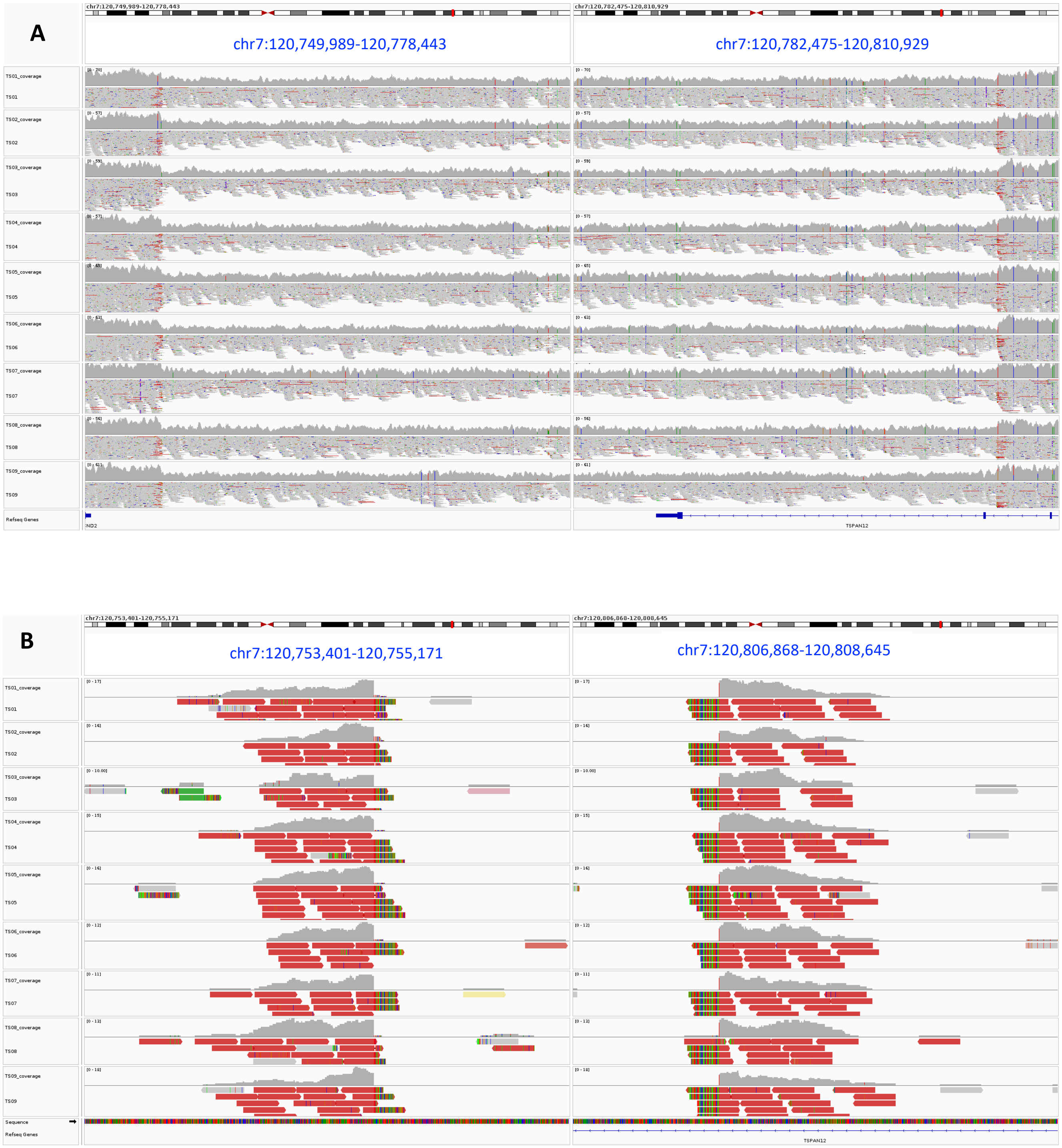

***Figure S2.* Shared haplotype surrounding the *TSPAN12* exon 7-8 deletion.** Black bar depicts the haplotype surrounding the *TSPAN12* deletion in TS01 (Case 3.1) and TS02 (Case 3.1's unaffected father). Grey bars indicate the genotypes are consistent with one haplotype matching that surrounding the deletion in TS01/2. The data corresponds to 1-Mb flanking each side of the *TSPAN12* deletion and shows variants with an allele frequency between 0.01 and 0.1 in gnomAD. Data is for GRCh38.

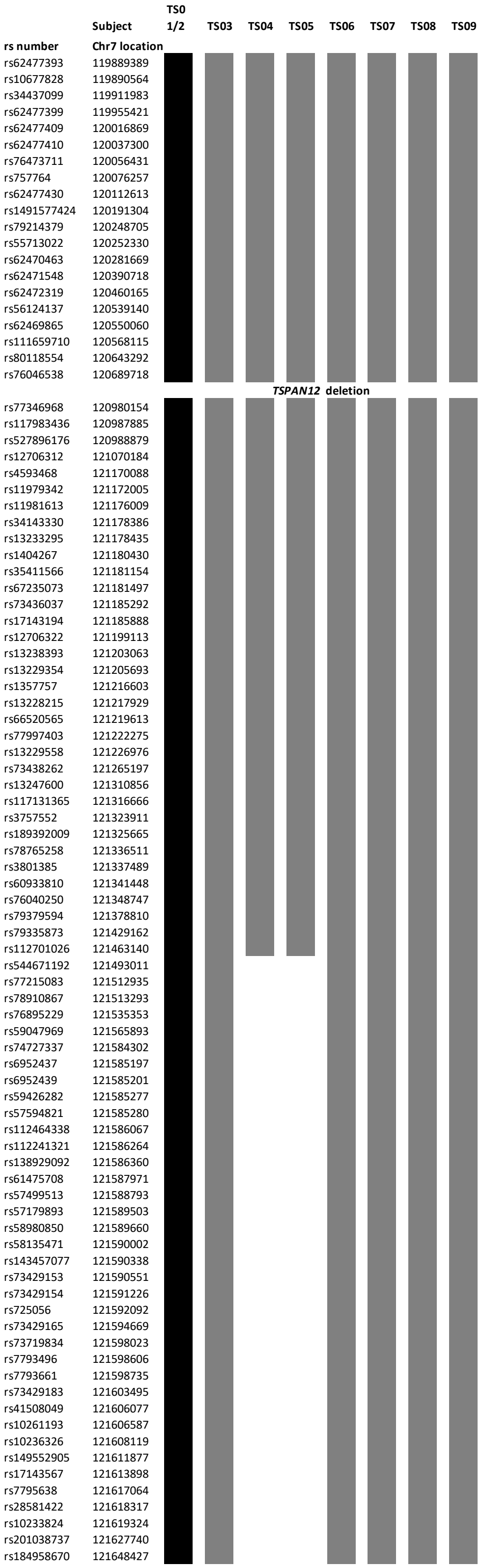

***Figure S3*. Conservation of amino acids predicted to be mutated in FEVR cases with *CTNND1* variants.** Alignments were created using the Constraint-based Multiple Alignment Tool (COBALT) (https://www.ncbi.nlm.nih.gov/tools/cobalt/). Protein sequences accession numbers: Human: NP_001078927.1, Dog: XP_005631252.1, Mouse: NP_001078919.1, Chicken: XP_015142330.1, Frog: NP_001119545.1, Zebrafish: XP_021332609.1.

```
                                 Gly87Arg
Human      72  DADLERRQKFSDLKLNGPQDHSHLLYSTIPRM 102
Dog        72  DADLERRQKFPDLKLNGPQDHSHLVYSTIPRM 102
Mouse      72  DADLERRQKFSDLKLNGPQDHNHLLYSTIPRM 102
Chicken    72  DADLERRQKFPDLKLNGPQDHSHLLYSTIPRM 102
Frog       56  DAELERRLKLSEGYINGTQY----------RM 76
Zebrafish  61  -----------------QQDGSPFLY-TSTRM 75

                                Ser312Phe
Human      299 MSDYGTARRTGTP--SDPRRRLRSYEDMIGE 327
Dog        299 MSDYGTARRTGTP--SDPRRRLRSYEDMIGE 327
Mouse      299 MSDYGTARRTGTP--SDPRRRLRSYEDMIGE 327
Chicken    299 MSDYGTARRAGTP--SDARRRLRSYEDMLVD 327
Frog       235 -----TGRRGANG--GDPRRRLRSYEDPLS- 257
Zebrafish  280 AMHYSTMPRLAHPHHAPPPRRTGSYEGTLDG 310

                                 Exon12del
Human      628 WFSRGKKPIEDPANDTVDFPKRTSPARGYELLFQP 662
Dog        628 WFSRGKKPTEDPANDTVDFPKRTSPARGYELLFQP 662
Mouse      628 WFSRGKKPTEDPANDTVDFPKRTSPARGYELLFQP 662
Chicken    629 WFSRGKKLPEDPGADTVDFPKRTTPAKGYELLFQP 663
Frog       557 ----GKKASEESVVDTIDFPKRTMPAQGYDLLFQP 585
Zebrafish  619 WFSKGKR-EDDGTSDTIDIPKRTTPAKGYELLFQP 652
```

***Figure S4. CTNND1* mutation spectrum.** Schematic representation of delta catenin showing domains and mutations. The protein domain information was obtained from UniProt (https://www.uniprot.org/). Variants previously reported in FEVR cases are in blue (Yang et al., 2022) and those in the current study are blue and underlined. Variants shaded in grey were reported in Non-Syndromic Cleft Lip with or without Cleft Palate cases (Cox et al., 2018). The variant shaded in black was reported in a cardiac malformation case (Zhao et al., 2023). Variants shaded in pink were reported in cases with craniofacial and cardiac syndrome (Alharatani et al., 2020). Variants shaded in green were found in cases with Blepharocheilodontic syndrome, in which the ones without italics were published by (Kievit et al., 2018) and variants with italics were published by (Ghoumid et al., 2017). Binding partners and location are shown in purple (Alharatani et al., 2020).

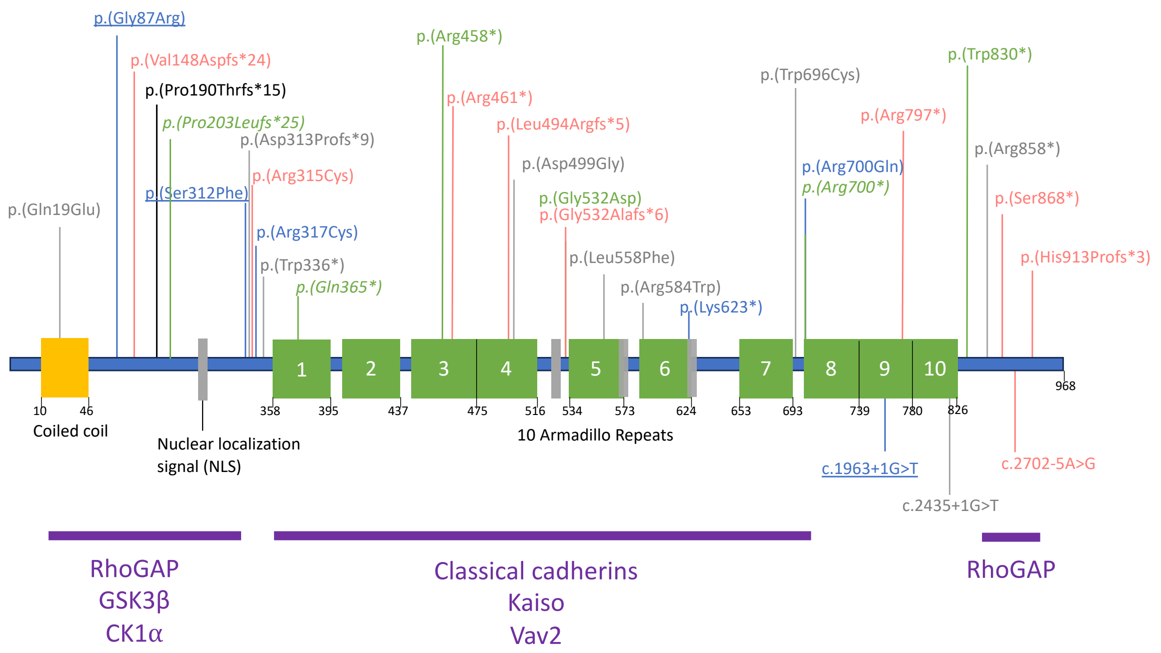

***Figure S5*. Frequency of HPO terms listed in the GEL FEVR cohort.** There are 47 different HPO terms submitted for the full FEVR cohort and the number of HPO terms submitted with each case ranges between 1-15, with an average of 5.7 per case. Thirty-one HPO terms were only assigned to single probands, 14 in solved cases and 16 in unsolved cases. However, 14 of the single entries were for eye phenotypes, including some which are associated with FEVR, eg. exudative vitreoretinopathy, rhegmatogenous retinal detachment, tractional retinal detachment, and vitreoretinopathy. When looking at the full cohort, the top 11 HPO terms are shown in the bar chart and include visual impairment, abnormal retinal vascular morphology, progressive visual loss, retinal exudate, abnormality of retinal pigmentation, constriction of peripheral visual field, retinal dystrophy, reduced visual acuity, non-progressive visual loss, retinal detachment and retinal fold. Many of these terms are quite generic (visual impairment) and only four are somewhat specific to FEVR (abnormal retinal vascular morphology, retinal exudate, retinal detachment, and retinal fold). No significant difference was observed between the top HPO terms in solved and unsolved cases (two tailed Fishers exact test). The corresponding P values are presented in the figure.

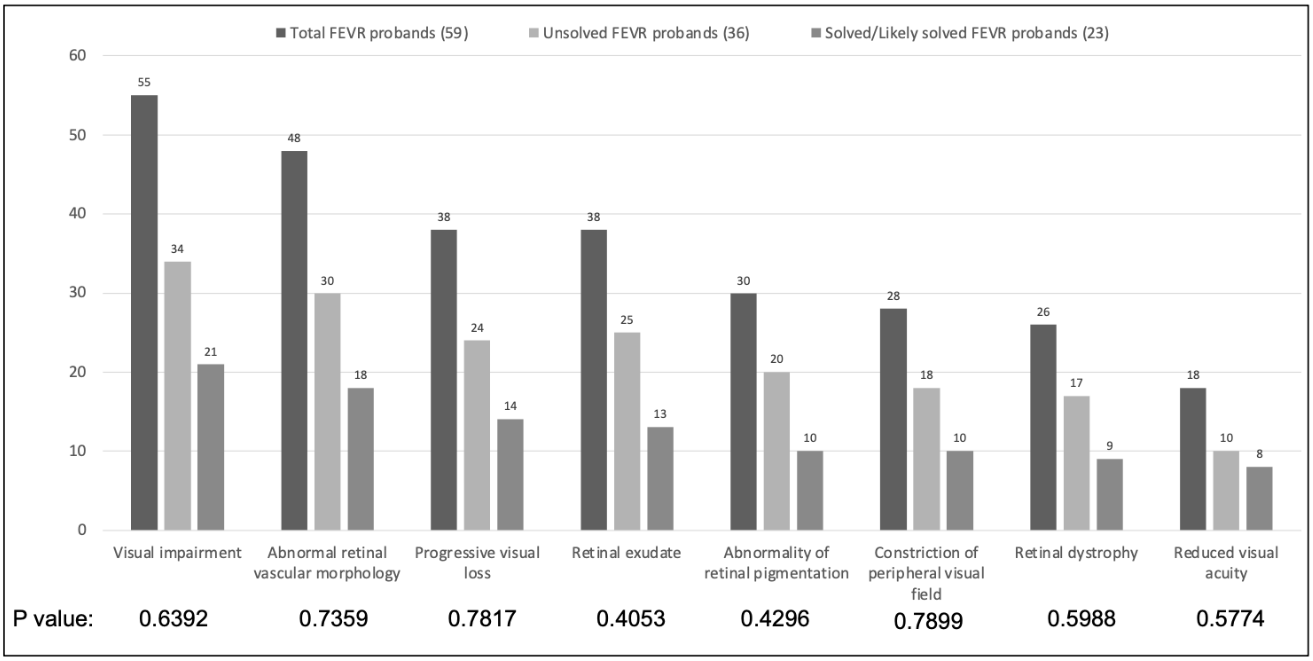

***Table S1*. Information for GEL participants containing *TSPAN12* exon7-8del.**

ID	Participant information	Ethnicity	HPO eye abnormality	Solved in GEL	Breakpoint (hg38)
TS01(Case 3.1)	FEVR	White: British	YES (visual impairment)	No	chr7:120754460-120807405del
TS02	Case 3.1's unaffected father	White: British	N/A	N/A	chr7:120754460-120807405del
TS03	Unaffected relative of GEL case	White: British	N/A	N/A	chr7:120754460-120807405del
TS04	Renal tract calcification (or Nephrolithiasis or nephrocalcinosis)	White: British	N/A	N/A	chr7:120754460-120807405del
TS05	TS04's affected sibling	White: British	N/A	N/A	chr7:120754460-120807405del
TS06	TS07's unaffected father	White: British	N/A	N/A	chr7:120754460-120807405del
TS07	Hereditary ataxia	White: British	YES (visual impairment)	No	chr7:120754460-120807405del
TS08	Breast cancer	Not stated	N/A	N/A	chr7:120754460-120807405del
TS09	Lung cancer	White: any other white background	N/A	NA	chr7:120754460-120807405del

***Table S2*. Summary of bioinformatics analyses undertaken to predict the pathogenic nature of the *CTNND1* missense variants.**

**Variant**	**AlphaMissense**	**Fathmm-XF**	**PolyPhen2**	**MutationTaster**	**SIFT**	**Blosum 62**	**ClinPred**	**PROVEAN**	**M-CAP**	**DANN**
p.(Gly87Arg)	Likely pathogenic (score 0.7143)	Deleterious (score 0.84287)	Possibly damaging (score 0.478)	Deleterious (prediction prob. 0.999519)	Deleterious (score 0)	Score -2	Damaging (score 0.705)	Neutral (score -1.66)	Deleterious (score 0.103)	Damaging (score 0.999)
p.(Ser312Phe)	Likely benign (score 0.3)	Deleterious (score 0.633132)	Benign (score 0.297)	Deleterious (prediction prob. 0.998088)	Tolerated (score 0.17)	Score -2	Damaging (score 0.796)	Neutral (score -1.89)	Deleterious (score 0.033)	Damaging (score 0.994)

The predictions are based on the translation of the MANE select transcript of *CTNND1* (NM_001085458.2). Across ten different pathogenic prediction tools, p.(Gly87Arg) was predicted as likely-pathogenic in nine and p.(Ser312Phe) was predicted as likely-pathogenic in six.

***Table S3*. Heterozygous variants of uncertain significance (VUS) detected in unsolved FEVR probands in genes reported to underlie FEVR.**

ID	Inheritance	Gene	GRCh38	Variant[1]	CADD[2]	SNP ID	Allele count in gnomAD[3]	Allele count in GEL
9.1	Unknown (single case)	*ILK*	11:6609768 A>G	c.901A>G: p.(Met301Val)	21.7	rs1339432987	4/1614192	2
27.1	Germline unaffected mother	*CTNNB1*	3:41240343 T>G	c.*1001T>G	22	-	Absent	2
19.1	Germline unaffected father	*EMC1*	1:19243651 C>T	c.343G>A: p.(Gly115Arg)	27.7	rs749646972	79/1614010	18
36.1	Germline unaffected mother	*EMC1*	1:19232647 A>G	c.1759T>C: p.(Cys587Arg)	25.6	rs201264850	10/1614158	2
21.1	Germline unaffected mother	*SNX31*	8:100600433 C>A	c.690G>T: p.(Gln230His)	23.2	rs368885001	36/1610112	4
35.1	Germline unaffected father	*ZNF408*	11-46702765 G>C	c.392G>C: p.(Ser131Thr)	26.9	rs765344131	25/1614046	7
55.1	Germline unaffected mother	*DLG1*	3:197091018 G>A	c.1555C>T: p.(Arg519Cys)	33	rs577786990	204/1596374	32

[1]HGVS annotation for transcripts *CTNNB1*: NM_001904.4; *ILK*: NM_004517.4; *EMC1*: NM_015047.3; *SNX31*: NM_152628; *DLG1*: NM_001366207 and *ZNF408*: NM_024741.3. [2]A scaled CADD score of 20 means that the variant is amongst the top 1% of deleterious variants in the human genome and a score of 30 means that the variant is in the top 0.1%. [3]The minor allele frequency (MAF) of the variants was obtained from gnomAD version v4.1.0.

SUPPLEMENTARY REFERENCES

Alharatani R, Ververi A, Beleza-Meireles A, *et al*. Novel truncating mutations in CTNND1 cause a dominant craniofacial and cardiac syndrome. Hum Mol Genet 2020;**29**:1900-1921. DOI:10.1093/hmg/ddaa050.

Cox LL, Cox TC, Moreno Uribe LM, *et al*. Mutations in the Epithelial Cadherin-p120-Catenin Complex Cause Mendelian Non-Syndromic Cleft Lip with or without Cleft Palate. Am J Hum Genet 2018;**102**:1143-1157. DOI: 10.1016/j.ajhg.2018.04.009.

Ghoumid J, Stichelbout M, Jourdain AS, *et al*. Blepharocheilodontic syndrome is a CDH1 pathway-related disorder due to mutations in CDH1 and CTNND1. Genet Med, 2017;**19**:1013-1021. DOI:10.1038/gim.2017.11.

Kievit A, Tessadori F , Douben H, *et al*. Variants in members of the cadherin-catenin complex, CDH1 and CTNND1, cause blepharocheilodontic syndrome. Eur J Hum Genet, 2018;**26**:210-219. DOI:10.1038/s41431-017-0010-5.

Yang M, Li S, Huang L, *et al*. CTNND1 variants cause familial exudative vitreoretinopathy through the Wnt/cadherin axis. JCI Insight 2022;**7**:e158428. DOI: 10.1172/jci.insight.158428.

Zhao X, Li X, Sun W, *et al*. Case report: "Major fetal cardiac pathology associated with a novel CTNND1 mutation". Front Pediatr, 2023;**11**:1180381. DOI: 10.3389/fped.2023.1180381.
